# The Appropriateness Criteria of Abdominal Fat Measurement at the Level of the L1-L2 Intervertebral Disc in Patients With Obesity

**DOI:** 10.3389/fendo.2021.784056

**Published:** 2021-12-14

**Authors:** Jing Sun, Han Lv, Meng Zhang, Mengyi Li, Lei Zhao, Na Zeng, Yawen Liu, Xuan Wei, Qian Chen, Pengling Ren, Yang Liu, Peng Zhang, Zhenghan Yang, Zhongtao Zhang, Zhenchang Wang

**Affiliations:** ^1^ Department of Radiology, Beijing Friendship Hospital, Capital Medical University, Beijing, China; ^2^ Department of General Surgery, Beijing Friendship Hospital, Capital Medical University & National Clinical Research Center for Digestive Diseases, Beijing, China; ^3^ National Clinical Research Center for Digestive Diseases, Beijing Friendship Hospital, Capital Medical University, Beijing, China; ^4^ School of Biological Science and Medical Engineering, Beihang University, Beijing, China

**Keywords:** upper abdominal MRI, single MR slice, obesity, abdominal adipose tissue, bariatric surgery

## Abstract

**Background:**

In this study, we proposed to use MR images at L1-L2 (lumbar) intervertebral disc level to measure abdominal fat area in patients with obesity. The quantitative results would provide evidence for the individualized assessment of the severity of obesity.

**Methods:**

All patients in the IRB-approved database of Beijing Friendship Hospital who underwent bariatric surgery between November 2017 and November 2019 were recruited. We retrospectively reviewed upper abdominal magnetic resonance (MR) data before surgery. We analyzed the correlation and consistency of the area of abdominal subcutaneous adipose tissue (ASAT) and visceral adipose tissue (VAT) measured at the L1-L2 and L2-L3 levels on MR images. We randomly distributed the cases into prediction model training data and testing data at a ratio of 7:3.

**Results:**

Two hundred and forty-five subjects were included. The ASAT and VAT results within the L1-L2 and L2-L3 levels were very similar and highly correlated (male^ASAT^: r=0.98, female^ASAT^: r=0.93; male^VAT^: r=0.91, female^VAT^: r=0.88). There was no substantial systematic deviation among the results at the two levels, except for the ASAT results in males. The intraclass correlation coefficients (ICCs) were 0.91 and 0.93 for male^ASAT^ and female^ASAT^, and 0.88 and 0.87 for male^VAT^ and female^VAT^, respectively. The ASAT/VAT area at the L2-L3 level was well predicted. The coefficient β of linear regression that predicted L2-L3 ASAT from L1-L2 ASAT was 1.11 for males and 0.99 for females. The R-squares were 0.97 and 0.91, respectively. For VAT prediction, the coefficient β was 1.02 for males and 0.96 for females. The R-squares were 0.82 and 0.77, respectively.

**Conclusion:**

For patients with obesity, the L1-L2 intervertebral disc level can be used as the substitution of L2-L3 level in abdominal fat measurement.

## Introduction

The number of patients with obesity worldwide has exceeded 200 million. The “obesity epidemic” has become a global public health problem (1, 2). Obesity can lead to type 2 diabetes mellitus (T2DM), coronary heart disease (CHD), nonalcoholic fatty liver disease (NAFLD) and other metabolic diseases, resulting in significant adverse effects on population survival outcomes (3, 4).

Obesity can be divided into several types, including abdominal obesity, which is more common among obese patients in Asia (5). Visceral fat accumulation is highly correlated with metabolic syndromes and high-risk cardiovascular diseases (6, 7). However, the anthropometric parameters, such as body mass index (BMI) and waist-hip ratio (WHR) only roughly evaluate abdominal adiposity (8). Liver acquisition with volume acceleration-flexible (LAVA⁃Flex) sequence of magnetic resonance (MR) imaging has been shown to be the optimal method for displaying and quantifying subcutaneous/visceral fat (5, 9). The accurate quantified fat area can provide evidence for the individualized assessment of the severity of obesity.

The volume measurement of total abdominal adipose tissue (TAAT) is highly demanding for image analysis and time-consuming, making it inappropriate for clinical application (6). To improve efficiency and reduce cost, researchers proposed to predict TAAT volume from a single axial slice. The choice of the standard single slice, however, remains controversial.

For patients with obesity, it is generally believed that the level of the L2-L3 intervertebral disc is a reliable anatomic site for quantifying abdominal adiposity (10–12). However, there are two significant shortcomings on L2-L3 level. First, the L2-L3 level generally do not include the liver and pancreas. The assessment of liver fat and pancreatic fat is valuable for the risk prediction of obesity-related diseases (9). Second, the upper abdominal MRI rarely includes the L2-L3 level (9). The upper abdominal MRI generally includes the L1-L2 level. If researchers enlarge the scanning range in order to cover L2-L3 level, it may take more than 20 seconds. It is difficult for patients to hold their breath during MR scanning, and significant motion artifacts will limit the measurement. Therefore, the L2-L3 level is not suitable for clinical use (9).

The L1-L2 level can well display both the liver and pancreas as well as subcutaneous and visceral fat. Several studies reported that VAT and ASAT measured at L1-L2 were also strongly correlated with total VAT volume (13, 14) and ASAT volume (11). Moreover, VAT within the L1-L2 level showed the best predictive ability for the risk of metabolic syndromes compared with other levels (13, 15). Given the better clinical appropriateness, a previous study with a large sample proposed that the L1-L2 level was the most appropriate axial level for the assessment of ASAT/VAT (5, 9). This new criterion still needs to be further validated by statistical evidence.

Thus, this study aimed to evaluate the appropriateness of the L1-L2 intervertebral disc level for the quantification of abdominal fat in patients with obesity. The hypothesis is that the L1-L2 level could serve as a reliable new standard for the abdominal fat measurement.

## Methods

### Subjects

According to the World Health Organization (WHO) criteria for obesity in the Asian population, the BMI cut-off point was set as ≥27.5kg/m^2^ for the diagnosis of morbid obesity (16). Participants in this study were recruited from patients who were diagnosed as obesity and underwent bariatric surgery in the Department of General Surgery in Beijing Friendship Hospital between November 2017 and November 2019. Anthropometric data were obtained from the Greater China Metabolic and Bariatric Surgery Database (GC-MBD), ClinicalTrials.gov ID: NCT03800160.

The inclusion criteria were as follows: (1) the upper abdominal MR scans included the LAVA-Flex sequence; (2) the level of the L1-L2 and L2-L3 intervertebral discs were both included; and (3) the ASAT at L1-L2/L2-L3 and (or) the VAT at L1-L2/L2-L3 were available for analysis.

The exclusion criteria were (1) patients with missing anthropometric data such as height or weight; (2) image artifacts limited ASAT or VAT measurement; (3) part of the image was lost (outside of the screen view); and (4) patients had contraindications to MR examination, such as claustrophobia, or metal implants, such as pacemakers and metal stents.

This study was approved by the Ethics Committees of Beijing Friendship Hospital (NO. 2017-P2-131-02). Informed consent was obtained from all participants.

**Figure 1:**
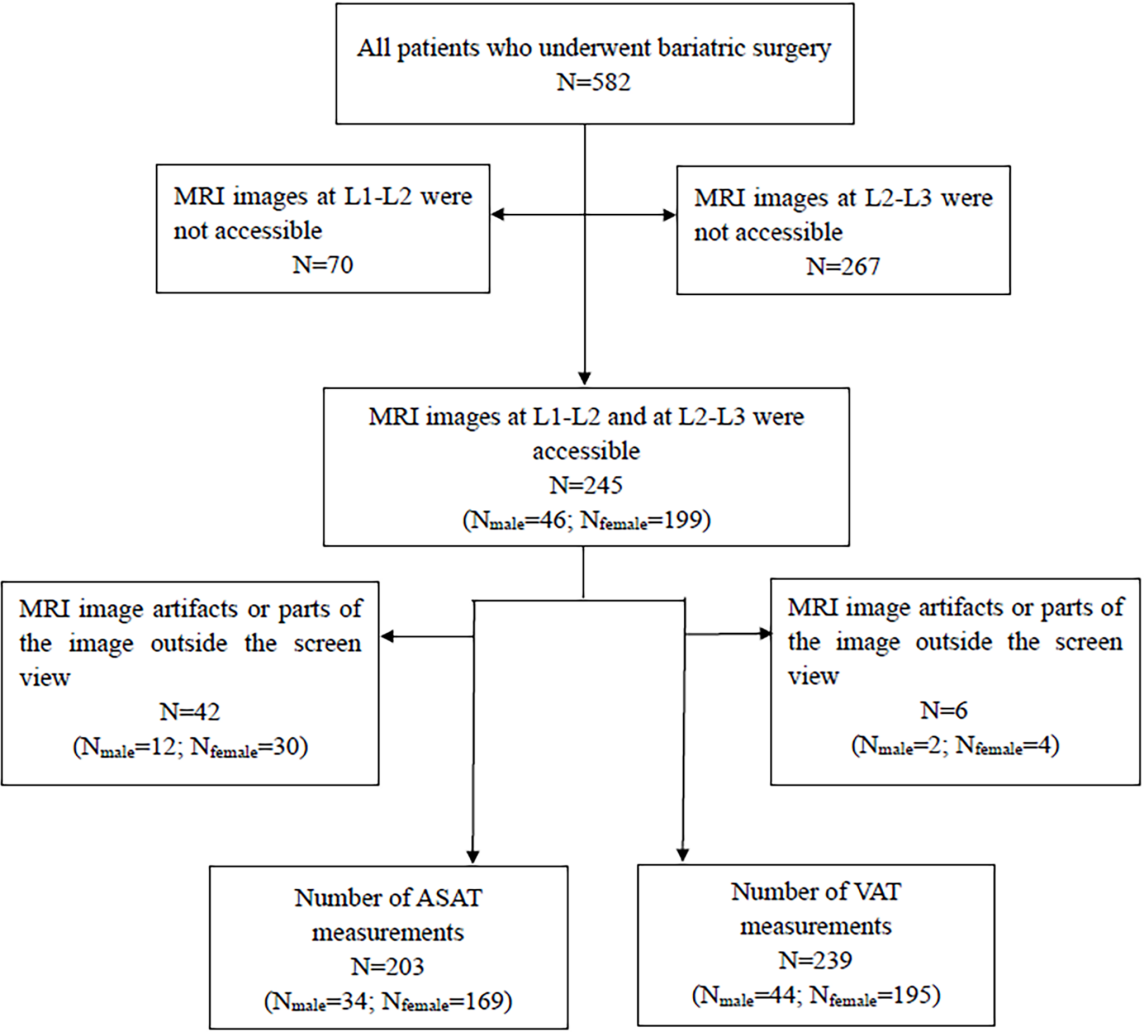
Enrolled patient selection.

Among the 582 patients who underwent bariatric surgery between November 2017 and November 2019, 70 were excluded because the upper abdominal MRI did not include the level of L1-L2, while 267 were excluded because MRI did not include the level of L2-L3. Thus, 245 (42.1%) patients were enrolled (46 males, 199 females) (**Figure 1**).

### MR Imaging Acquisition

All enrolled patients without contraindications were recommended to perform upper abdominal MR examination for preoperative evaluation. The examination complied with the clinical routine imaging examination norms. The MR images were collected by a Discovery MR 750 W 3.0T MR scanner (General Electric (GE) Medical Systems, Milwaukee, WI, USA). The coil used was an eight-channel phased-array abdominal coil. The scanning range was from the top of the diaphragm to the level of the renal hilum.

**Figure 2:**
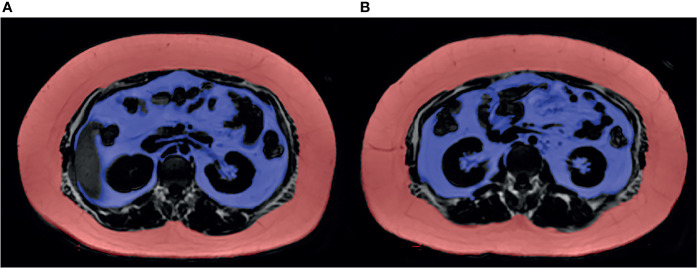
Fat LAVA-Flex image clearly showed subcutaneous and visceral adipose tissue at the level of the L1-L2 and L2-L3 intervertebral discs. **(A)** The ASAT area measured at L1-L2 was 42,973 mm^2^, and the VAT area was 19,087 mm^2^. **(B)** The ASAT area measured at L2-L3 was 43,510 mm^2^, and the VAT area was 17,591 mm^2^. The ASAT area was marked in red. The VAT area was marked in purple.

LAVA-Flex sequences were collected, and the corresponding lipid phase diagrams, Fat LAVA-Flex, were calculated for the measurement of ASAT and VAT. The acquisition parameters were TR=4.1 ms; TE=1.9 ms; FOV=40 cm×32 cm; slice thickness=4 mm; matrix=160×160; flip angle=12°; NEX=1; and acquisition time=15 s. Then, the cross-sectional images at the L1-L2 and L2-L3 intervertebral discs were exported and collected through the image export function of RadiAnt DICOM Viewer (version 2020.2, Medixant, Poznan, Poland). Finally, two radiologists with 5 years of experience manually delineated the ROIs of ASAT and VAT on the collected L1-L2 and L2-L3 images through ITK-SNAP software (version 3.8.0). The ASAT was distinguished from VAT by the exterior margin of the abdominal wall muscle. The ROIs of ASAT and VAT were shown in **Figure 2**. The intraclass correlation coefficients (ICCs) between radiologists were 0.998 and 0.989 for ASAT and VAT, respectively (both *p*<0.001).

To eliminate the influence of single slice thickness, the measured ASAT and VAT volumes (mm^3^) were converted into corresponding areas (mm^2^). The conversion formula was:


Area=voxel count×pixel spacing (x)×pixel spacing (y)

### Statistics

The Kolmogorov-Smirnov test was applied to measure normality of data. Data with a normal distribution were represented by the mean ± standard deviation, while those with a skewed distribution were represented by the median (P_25_, P_75_). The paired *t* test and Wilcoxon test were used to analyze whether there were differences in ASAT and VAT area, respectively, between the level of L1-L2 and L2-L3 for males and females.

According to the data distribution, Pearson correlation or Spearman correlation was used to analyze the internal relationship between ASAT/VAT measured at L1-L2 and at L2-L3. The results were presented with scatterplots. Second, we investigated the agreement between ASAT/VAT measured at L1-L2 and at L2-L3 by Bland-Altman diagrams. The ICCs were also calculated.

The enrolled patients were randomly distributed into prediction model training and model testing at a ratio of 7:3. First, a linear regression model was trained to predict ASAT/VAT at L2-L3 from ASAT/VAT at L1-L2. Second, we substituted ASAT/VAT values at L1-L2 into the regression formula to obtain the predicted values of ASAT/VAT at L2-L3 separately for males and females. Finally, the correlation between predicted and actual ASAT/VAT area at L2-L3 was estimated by Pearson correlation.

## Results

### Characteristics of Enrolled Patients

The demographic data of enrolled patients at baseline were shown in **Table 1**. Thirty-four males and one hundred and sixty-nine females had ASAT area measurements at both the L1-L2 level and the L2-L3 level, while forty-four males and one hundred and ninety-five females had VAT area measurements at both levels.

**Table 1:** Baseline data of 245 patients who underwent bariatric surgery.

	All patients (n = 245)	Male (n = 46)	Female (n = 199)
Age (Y)	31.0 (26.0,37.0)	31.7 ± 7.0	31.0 (26.0,37.0)
Weight (kg)	103.7 (91.4,119.5)	130.8 ± 26.2	101.9 ± 18.5
BMI (kg/m^2^)	37.4 (32.6,41.9)	41.7 ± 7.4	36.7 (32.4,41.0)
L1-L2 ASAT (×10^4^ mm^2^)	3.0 (2.3,4.0)	3.2 ± 1.3	3.0 (2.2,3.9)
L1-L2 VAT (×10^4^ mm^2^)	1.8 (1.5,2.3)	2.8 ± 0.7	1.7 ± 0.5
L2-L3 ASAT (×10^4^ mm^2^)	3.4 (2.6,4.4)	3.7 ± 1.5	3.2 (2.5,4.2)
L2-L3 VAT (×10^4^ mm^2^)	1.8 (1.4,2.2)	2.6 ± 0.7	1.6 ± 0.5

BMI, body mass index; L, lumber; ASAT, abdominal subcutaneous adipose tissue; VAT, visceral adipose tissue.

ASAT area was larger at the L2-L3 level than at the L1-L2 level (average area in males: 3.7×10^4^ mm^2^ vs 3.2×10^4^ mm^2^, *p*<0.001; median area in females: 3.2×10^4^ mm^2^ vs 3.0×10^4^ mm^2^, *p*<0.001). In both sexes, VAT area at the L1-L2 level was greater than that at the L2-L3 level (average area in males: 2.8×10^4^ vs 2.6×10^4^ mm^2^, *p*=0.001; average area in females: 1.7×10^4^ vs 1.6×10^4^ mm^2^, *p*<0.001). The results were presented in **Table 1**.

### Correlation and Agreement Between ASAT/VAT at L1-L2 and L2-L3

**Figure 3:**
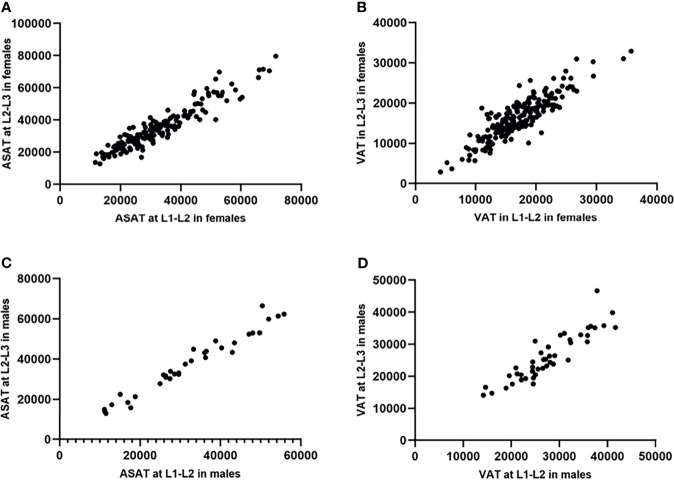
Scatterplots showed an approximately linear distribution of ASAT and VAT at L1-L2/L2-L3 in females and males (measurement unit: mm^2^). **(A)** The ASAT measured at L1-L2/L2-L3 in females were highly correlated. r=0.93. **(B)** The VAT measured at L1-L2/L2-L3 in females were highly correlated. r=0.88. **(C)** The ASAT measured at L1-L2/L2-L3 in males were highly correlated. r=0.98. **(D)** The VAT measured at L1-L2/L2-L3 in males were highly correlated. r=0.91.

ASAT measured at the L1-L2 and L2-L3 levels and VAT measured at the two levels were confirmed to be highly correlated (male^ASAT^: r=0.98, *p*<0.001, female^ASAT^: r=0.93, *p*<0.001; male^VAT^: r=0.91, *p*<0.001, female^VAT^: r=0.88, *p*<0.001). Scatterplots showed an approximately linear distribution of ASAT and VAT at L1-L2 and L2-L3 (**Figure 3**).

**Figure 4:**
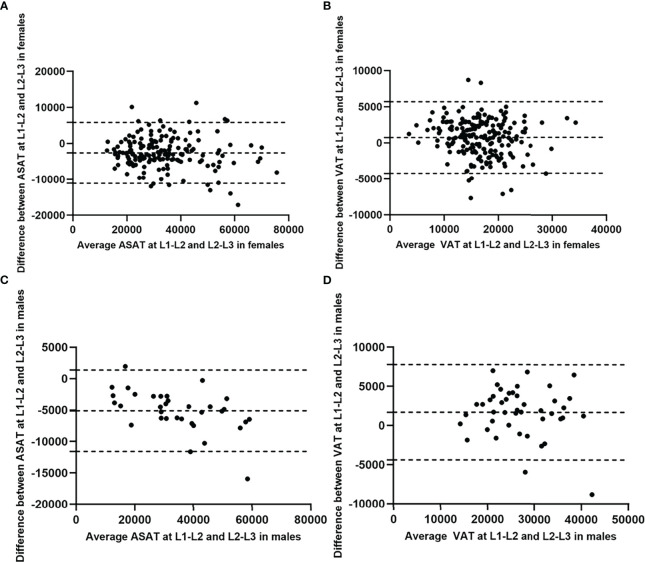
Bland-Altman diagrams showed the agreement between ASAT/VAT measured at L1-L2 and at L2-L3 in females and males (measurement unit: mm^2^). The Bland-Altman plots showed no substantial systematic deviation among ASAT and VAT results at L1-L2/L2-L3 in females **(A, B)**, and VAT results at L1-L2/L2-L3 in males **(D)**. There was a systematic deviation between ASAT measurements at L1-L2 and at L2-L3 in males **(C)**, given the mean difference was far away from 0.

The Bland-Altman plots showed no substantial systematic deviation among ASAT and VAT results at L1-L2/L2-L3 in females (**Figures 4A, B**), and VAT results at L1-L2/L2-L3 in males (**Figure 4D**). There was a systematic deviation between ASAT measurements at L1-L2 and at L2-L3 in males (**Figure 4C**), given the mean difference was far away from 0. The ICCs (single measures) were 0.91 (95% CI 0.12-0.98, *p*<0.001) and 0.93 (95% CI 0.82-0.96, *p*<0.001) for male^ASAT^ and female^ASAT^, and 0.88 (95% CI 0.73-0.94, *p*<0.001) and 0.87 (95% CI 0.82-0.90, *p*<0.001) for male^VAT^ and female^VAT^.

### L2-L3 ASAT/VAT Prediction Model Based on L1-L2 ASAT/VAT

**Figure 5:**
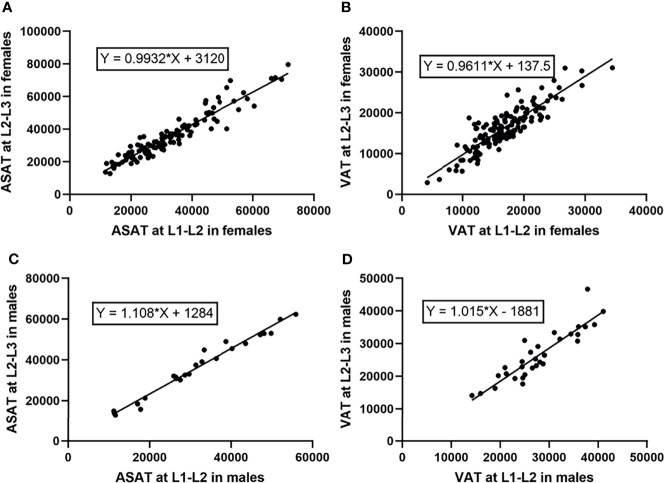
Linear regression models predicting ASAT and VAT area at the L2-L3 level from those at the L1-L2 level (measurement unit: mm^2^). **(A)** The linear regression model predicting ASAT area at L2-L3 from those at L1-L2 in females. The β coefficient was 0.99. **(B)** The linear regression model predicting VAT area at L2-L3 from those at L1-L2 in females. The β coefficient was 0.96. **(C)** The linear regression model predicting ASAT area at L2-L3 from those at L1-L2 in males. The β coefficient was 1.11. **(D)** The linear regression model predicting VAT area at L2-L3 from those at L1-L2 in males. The β coefficient was 1.02.

The β coefficients of the linear regression models that predicted ASAT area at the L2-L3 level from ASAT at the L1-L2 level were 1.11 (males, 95% CI 1.03-1.19, *p*<0.001) and 0.99 (females, 95% CI 0.93-1.05, *p*<0.001). The R-squares were 0.97 and 0.91 for males and females, respectively (**Figure 5**).

For VAT prediction, the β coefficients were 1.02 (males, 95% CI 0.83-1.20, *p*<0.001) and 0.96 (females, 95% CI 0.87-1.05, *p*<0.001). The R-squares were 0.82 and 0.77 for males and females, respectively (**Figure 5**).

**Figure 6:**
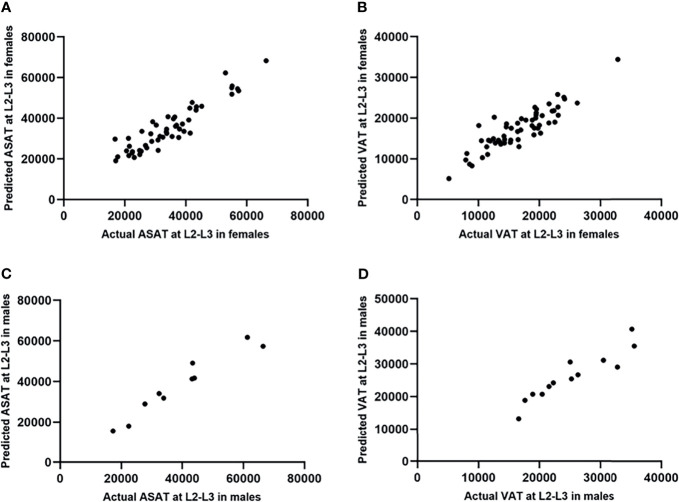
Scatterplots showed the testing results of the prediction model of ASAT/VAT area at L2-L3 (measurement unit: mm^2^). **(A)** The actual and predicted ASAT area at L2-L3 in females were highly correlated. r=0.93. **(B)** The actual and predicted VAT area at L2-L3 in females were highly correlated. r=0.89. **(C)** The actual and predicted ASAT area at L2-L3 in males were highly correlated. r=0.97. **(D)** The actual and predicted VAT area at L2-L3 in males were highly correlated. r=0.93.

Scatterplots were drawn with the actual and predicted ASAT/VAT at the L2-L3 level as the horizontal and vertical coordinates, respectively (**Figure 6**). The results showed that the ASAT values were highly correlated, with correlation coefficients of 0.97 in male patients (*p*<0.001) and 0.93 in female patients (*p*<0.001). The correlation between the actual and predicted VAT at L2-L3 was slightly weaker than that of ASAT, with correlation coefficients of 0.93 in males (*p*<0.001) and 0.89 in females (*p*<0.001).

## Discussion

Our study confirmed that ASAT and VAT results within L1-L2 and L2-L3 levels were highly correlated. The ASAT/VAT area within L2-L3 level could be well predicted by the ASAT/VAT area within L1-L2 level. Therefore, the L1-L2 level can be used as the substitution of L2-L3 level.

However, we also found the higher correlation and higher agreement in ASAT group than in VAT group. This may be due to the composition of VAT. VAT consists of two components, intraperitoneal adipose tissue (IPAT) and extraperitoneal adipose tissue (EPAT) (11, 17). EPAT mainly acts as a fat pad to provide mechanical support for the retroperitoneal organs, with little change among abdominal axial slices. In contrast, IPAT, which mainly distribute in the upper abdomen, has a high metabolic activity (17). This may be part of the reason for the larger difference in VAT area between different levels.

In previous studies, the L2-L3 level received more attention. But this study confirmed that, patients with large body size had a lower L2-L3 inclusion rate (245/582, 42.1%), compared to a higher L1-L2 inclusion rate (512/582, 88.0%). 267 patients (45.9%) were excluded for not including L2-L3 level, whereas 70 patients (12.0%) were excluded for not including L1-L2 level. This finding further demonstrated the necessity of applying L1-L2 level as the standard site for abdominal fat measurement. The study by Lv et al. using upper abdominal MR showed 93.1% of patients with the L1-L2 level available and 43.7% with the L2-L3 level available (9). Their results provided reliable proof of our conclusion.

In the previous study, we compared the displaying capability towards adipose tissue on several abdominal MR sequences, including fat LAVA-Flex, PDFF, and in-phase/out-of-phase T1WI, and fat-suppressed T1WI and T2WI. The study found that the LAVA-Flex sequence had a high imaging resolution. Moreover, on the fat images of LAVA-Flex sequence, the adipose tissue presented a significantly high signal, which can be easily distinguished from abdomen viscera and blood vessels. Thus, the margin of ASAT/VAT could be well recognized manually or automatically. Other sequences were not suitable for quantifying ASAT/VAT. Therefore, the fat LAVA-Flex image was considered the best choice to quantify adipose tissue (9).

The upper abdominal MR examination is a highly effective one-stop measurement. The collection time of LAVA-Flex sequence takes about 15 seconds. At the L1-L2 level, the researchers are able to precisely evaluate hepatic and pancreatic fat deposits, and ASAT/VAT before and after surgery. These measurements are valuable information for the risk prediction of obesity-related diseases (9). MR can also be used to analyze the fatty acids (FA) composition in different adipose tissues (18). The MR assessment of fat deposition in the vertebra is also widely used in clinic and in research projects (19). Moreover, a MR examination only costs about 120 dollars in China. Considering the cost of MR examination and the precise quantified information it provides, it is of great value to perform MR examinations in patients with obesity.

Beyond MR, QCT or automatic segmentation software based on CT images can achieve automatic or manual quantification of ASAT/VAT (10, 20). QCT is also the gold standard for bone mineral density measurement (21). But QCT has the deficiency of exposing to ionizing radiation. It is unable to quantify the liver fat fraction either. DXA can be used to measure visceral fat (22). The radiation dose of DXA is lower than that of CT, but it requires an osteo-densitometer. The measurement of hepatic, pancreatic and subcutaneous fat by DXA is not accurate enough (23). Ultrasonography can assess adipose tissue thickness, but cannot directly quantify the area or volume of ASAT/VAT.

In clinical work or research projects, the upper abdominal MR examination should include the axial 3D DIXON, axial PDFF, coronal T2WI, and axial fat-saturated T2WI sequences. The 3D DIXON technology is a mature water-fat separation imaging technology, including LAVA-Flex (GE), VIBE-DIXON (Siemens), and FFE-mDIXON (Philips). The fat image of DIXON sequence can well recognize adipose tissue, and thus is best for ASAT/VAT quantification (9). If the 3.0 T MR device is not available, the 2D dual-echo sequence on 1.5 T MR can be applied as an alternative. The PDFF sequence is essential for the measurement of hepatic PDFF and can accurately assess the hepatic steatosis grade (24). Coronal T2WI and axial T2WI+FS sequences can provide information about occasional but important diseases, such as gallstones and hiatal hernia, etc.

This study did not deny the reliability of L2-L3 level, but L1-L2 level could be a good candidate as well. Each institution can choose the evaluation level according to its own conditions. The MR examination is also not mandatory or a necessity. If there is a demand for fat area quantification, the application of upper abdominal MR with the inclusion of L1-L2 level is prior to be considered. The FOV should be large enough to cover the entire abdominal skin.

For patients, the distribution of adipose tissue may be varied for different patients with the same BMI (25). The quantitative results are valuable evidence to help clinicians decide the surgical procedure. Moreover, the assessment of abdominal fat distribution in patients with adolescent obesity could provide information for treatment decisions. The patients and clinicians may also witness the curve of fat loss according to the quantified results (26). For researchers, the quantified area of abdominal fat can be used to evaluate the risk of obesity-related disease, predict the outcome, or for other purposes of research.

### Limitations

One of the limitations was that the data were from a single center from Chinese population, which is one of the largest-volume centers of bariatric and metabolic surgery in China. Second, the number of male patients enrolled was relatively small, As the majority of patients receiving bariatric surgery at our center were females (73.3%). Third, whether MR examination can be performed on patients with a large waist circumference (horizontal diameter > 70 cm) or body weight > 135-200 kg should be carefully evaluated (27). An MR scanner with a larger aperture (e.g., MR with an aperture of 70 cm or greater) could be used if necessary. Weight is not an absolute exclusion criterion.

## Conclusion

For patients with obesity, the L1-L2 intervertebral disc level can be used as the substitution of L2-L3 intervertebral disc level when measuring abdominal fat. We recommend the application of upper abdominal MR with the inclusion of L1-L2 level when there is a demand for fat area quantification.

## Data Availability Statement

The raw data supporting the conclusions of this article will be made available by the authors, without undue reservation.

## Ethics Statement

Informed consent was obtained from all individual participants included in the study. This study was performed in accordance with the principles of the Declaration of Helsinki and was approved by the Ethics Committees of Beijing Friendship Hospital, Capital Medical University.

## Author Contributions

Conception and design: JS and HL. Administrative support: PZ, ZY, ZZ, and ZW. Provision of study materials or patients: ML, MZ, YanL, PZ, and ZZ. Collection and assembly of data: JS, HL, and LZ. Data analysis and interpretation: JS, HL, LZ, YawL, NZ, XW, QC, and PR. Manuscript writing: JS and MZ. All authors contributed to the article and approved the submitted version.

## Funding

This work was supported by Beijing Scholars Program [No. (2015) 160].

## Conflict of Interest

The authors declare that the research was conducted in the absence of any commercial or financial relationships that could be construed as a potential conflict of interest.

## Publisher’s Note

All claims expressed in this article are solely those of the authors and do not necessarily represent those of their affiliated organizations, or those of the publisher, the editors and the reviewers. Any product that may be evaluated in this article, or claim that may be made by its manufacturer, is not guaranteed or endorsed by the publisher.
